# Addition of exenatide twice daily to basal insulin for the treatment of type 2 diabetes: clinical studies and practical approaches to therapy

**DOI:** 10.1111/ijcp.12032

**Published:** 2012-10-14

**Authors:** G S Tobin, M K Cavaghan, B J Hoogwerf, J B McGill

**Affiliations:** 1 Division of Endocrinology, Metabolism & Lipid Research, Washington University in St. LouisSt. Louis, MO, USA; 2 Division of Endocrinology and Metabolism, School of Medicine, Indiana UniversityIndianapolis, IN, USA; 3 Eli Lilly and Co.Indianapolis, IN, USA

## Abstract

**Background:**

Type 2 diabetes is a progressive disease that requires stepwise additions of non-insulin and insulin therapies to meet recommended glycaemic goals. The final stage of intensification may require prandial insulin, adding complexity and increased risks of hypoglycaemia and weight gain.

**Aims:**

This review assesses the benefits and risks of adding exenatide twice daily, a glucagon-like peptide 1 receptor agonist, in patients with type 2 diabetes who are currently treated with basal insulin, but have failed to reach their glycaemic goals.

**Methods and Results:**

Based on data from published studies, exenatide has a number of actions that complement basal insulin therapy. Exenatide has been shown to increase glucose-dependent insulin production, suppress abnormal plasma glucagon production, slow gastric emptying, enhance liver uptake of glucose and promote satiety. A recently published randomised clinical trial reported that the addition of exenatide twice daily to titrated basal insulin provided greater glycaemic control than titrated basal insulin alone, and did so without an increase in hypoglycaemic events and with modest weight loss. Exenatide use was associated with gastrointestinal side effects. The recent randomised trial confirmed and extended data from a number of prior observational studies that demonstrated the efficacy and safety of insulin/exenatide combination therapy. Practical considerations for adding exenatide twice daily to ongoing basal insulin are discussed.

**Review criteria**

PubMed and the Cochrane Central Register of Controlled Trials were searched for clinical trials that had the words ‘exenatide’ and ‘insulin’ in the title or abstract. Retrieved studies were examined on a case-by-case basis to identify those describing insulin/exenatide combination therapy. In addition, general information was gathered from the literature published on PubMed (until 01 February 2012) on the topics of type 2 diabetes, exenatide and insulin.

**Message for the clinic**

In patients with type 2 diabetes who have failed to reach glycaemic goals, studies suggest that adding exenatide twice daily to actively titrated basal insulin results in robust reductions in HbA1c, modest weight reduction, no significant increase in hypoglycaemia and a possible reduction in insulin dose. The combination appears to be efficacious across a range of patient types. Several titration regimens are provided in this review, along with recommendations for down-titration of insulin in patients with minimally elevated fasting plasma glucose levels or an HbA1c < 8%.

## Introduction

In patients with type 2 diabetes, the goals of pharmacotherapy are to reduce the risks of microvascular and macrovascular complications by achieving glucose values near the normal range, consistent with glycated haemoglobin (HbA1c) targets. Consensus statements by major organisations recommend HbA1c levels at or below 7% (1–3) or 6.5% (4). As blood glucose levels tend to rise over time despite pharmacotherapy (5), most patients require periodic intensification of therapy as the disease progresses. In addition to lifestyle modification, the consensus treatment algorithm from the American Diabetes Association (ADA) and the European Association for the Study of Diabetes (EASD) recommends a stepwise approach to therapeutic intensification (3).

Even after addition of insulin, many patients fail to reach therapeutic targets (6–11). Moreover, insulin therapy is associated with iatrogenic hypoglycaemia (12) and weight gain (13). These concerns have generated recent commentary on optimum management strategies for type 2 diabetes (14,15). This review examines the benefits and risks of adding exenatide twice daily, a glucagon-like peptide 1 (GLP-1) receptor agonist, in patients with type 2 diabetes who are currently treated with basal insulin, but have failed to reach their glycaemic goals. Data from a randomised trial reported by Buse et al. (16) and several observational studies that evaluated the combination of insulin and exenatide (17–24) are reviewed to provide the basis for practical approaches to implement therapy with these two pharmacological agents.

## Rationale for combining exenatide twice daily and basal insulin

**Figure 1:**
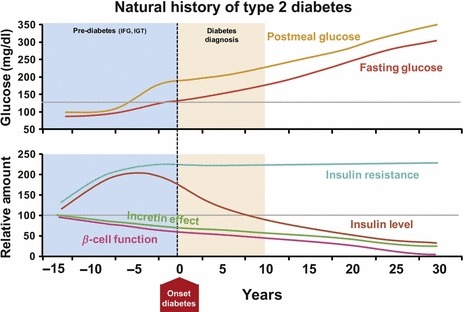
Natural history of type 2 diabetes. Representative depiction of the natural history of type 2 diabetes. Both the time course and relative function are descriptive. IFG, impaired fasting glucose; IGT, impaired glucose tolerance. For glucose, 1 mg/dl = 0.5551 mmol/l (25)

Type 2 diabetes is characterised by a progressive increase in fasting and postprandial plasma glucose concentrations (Figure 1) (25,26). Worsening hyperglycaemia in type 2 diabetes may result from a number of inter-related pathologies, including decline in beta-cell function, insulin resistance, increased hepatic glucose production associated with inappropriately high levels of glucagon and reduced GLP-1 production. This multi-system involvement and complex progressive pathophysiology support the concept that multi-drug treatment, which targets multiple functional defects of the disease, may be necessary for maintenance of optimal glycaemic control as the disease advances (26). Consequently, the current ADA/EASD treatment algorithm recommends the use of multiple antihyperglycaemic drugs with different mechanisms of action to maximise therapeutic benefit (3).

Based on its mechanism of action, exenatide is a pharmacological agent that should complement the actions of basal insulin. Mammalian GLP-1, a hormone released from the intestinal mucosa during meals, normally functions to reduce postprandial blood glucose via stimulation of glucose-dependent insulin secretion and suppression of glucagon (27). Exenatide has 53% amino-acid sequence homology with native human GLP-1 (27) and recapitulates many of its antihyperglycaemic activities in patients with type 2 diabetes (28–31). Thus, exenatide increases insulin production and secretion in response to hyperglycaemia, suppresses inappropriate plasma glucagon secretion, slows gastric emptying and decreases hepatic glucose production, all of which combine to lower blood glucose. In the US, exenatide twice daily (Byetta®, Amylin Pharmaceuticals, LLC) is indicated for subcutaneous administration prior to the two main meals of the day for the treatment of type 2 diabetes in patients with inadequate glucose control (32). Exenatide twice-daily treatment was primarily associated with a significant reduction in prandial glucose, whereas basal insulin was predominantly associated with reduced fasting plasma glucose (FPG) (33,34). Thus, evidence suggests that exenatide twice daily and basal insulin may have naturally complementary effects on glucose control.

The safety profile of exenatide therapy was exhaustively evaluated in a recent integrated safety analysis of pooled data from 19 completed randomised controlled clinical trials (35). In total, the meta-analysis examined 5594 study patients (3261 on exenatide; 2333 on comparators) who had received, on average, 166–171 days of study drug exposure. Relatively, few patients discontinued from the trials because of an adverse event (8% of patients on exenatide and 2% in the pooled comparator group). The most common adverse events in patients on exenatide were gastrointestinal in nature and included nausea [37% for exenatide vs. 8% for comparators; risk difference (RD) 29% (95% CI, 26.6–30.6)], vomiting [14% vs. 3%; RD 11% (95% CI, 9.3–12.0)] and diarrhoea [11% vs. 5%; RD 5% (95% CI, 3.8–6.6)]. The incidence of nausea and vomiting decreased substantially with increasing time on exenatide. The incidence of hypoglycaemia (minor or major) with concomitant sulfonylurea (exenatide 26.5%, pooled comparator 20.7%) was higher than that without sulfonylurea (exenatide 3.1%, pooled comparator 2.7%). Serious adverse events, discontinuations because of serious adverse events and deaths were reported with similar frequency in the exenatide and pooled comparator groups. Composite exposure-adjusted incidence rates were not statistically different between groups for pancreatitis, renal impairment or major adverse cardiac events; there was a difference in incidence rates for benign thyroid neoplasm (0.3% vs. 0%).

In the USA, exenatide twice daily is approved for use with insulin glargine in patients requiring additional glycaemic control, based on the study by Buse and colleagues (16), whereas in the European Union, exenatide twice daily is approved for use with other basal insulins (including neutral protamine Hagedorn insulin). The clinical approaches proposed in this article focus exclusively on the addition of exenatide to insulin glargine. Exenatide has not been approved for use with insulin mixtures or any short-acting insulins and, thus, no guidance about such combinations is provided.

## Exenatide twice daily and insulin combination therapy in type 2 diabetes: clinical studies

Buse et al. (16) performed a prospective, parallel, randomised, placebo-controlled trial that compared exenatide twice-daily injections or placebo added with actively titrated insulin glargine (Table 1). A key feature of the trial was its use of a validated insulin intensification algorithm based on the regimen described in the earlier Treat-to-Target Trial (10). The algorithm mandated insulin titration according to a simple regimen that targets FPG concentrations to ≤ 100 mg/dl (5.6 mmol/l) (Table 2). The methods and results in the Buse et al. study (16) therefore demonstrated not only the efficacy and safety of titrated insulin glargine in combination with exenatide in a clinical trial setting, but also can be used to support the practical approaches recommended in the next section.

**Table 1:** Insulin / exenatide combination studies

	Baseline values					
		
	Patient age (years)*	T2DM Duration (years)*	BMI (kg/m^2^)*	HbA1c (%)*	Total Insulin Dose (U/day)*	Description/outcomes
Buse et al. [16] (2011)	59 ± 9†	12 ± 7†	33.8 ± 5.8†	8.32 ± 0.85†	49.5 ± 29.9†	Prospective randomised placebo-controlled trial comparing glargine + exenatide (*n *= 137) vs. glargine + placebo (*n *= 122) Follow up, 30 weeks HbA1c decreased by 1.74% with glargine + exenatide vs. 1.04% with glargine + placebo Weight decreased by 1.8 kg with glargine + exenatide, increased by 1.0 kg with glargine + placebo Insulin increased 13 U/day with glargine + exenatide vs. 20 U/day with glargine + placebo Estimated rate of minor hypoglycaemia was similar between groups
Levin et al. [17] (2011)	60 ± 9.5‡	8.0 ± 5.6 –11.8 ± 7.1‡	37.6 ± 6.7‡	8.8 ± 1.3‡	0.45 ± 0.32§	Retrospective US chart review comparing different orders of addition (glargine + exenatide [*n *= 121] vs. exenatide + glargine [*n *= 44]) Follow up, 24 months HbA1c decreased by 0.7% with glargine + exenatide and 1% with exenatide + glargine Weight decreased by 2.5 kg in the glargine + exenatide group, increased by 0.7 ± 8.3 kg in the exenatide + glargine Hypoglycaemia was similar in the two treatment groups
Thong et al. [18] (2011)	55.8 ± 10.4¶	11.0¶	40.4 ± 7.8¶	9.52 ± 1.72¶	118 ± 104¶	Prospective nationwide (UK) audit, including 1257 patients who received exenatide as add-on to insulin Follow up, 12 months Addition of exenatide to patients continuing insulin resulted in: HbA1c reduction of 0.5%Weight reduction of 5.8 kgAn insulin dose reduction of 42 units per day, and an insulin discontinuation rate of 16.6%Patients receiving both exenatide + insulin experienced more hypoglycaemia than those receiving exenatide without insulin
Arnolds et al. [19] (2010)	56 ± 6**	6 ± 2**	31.2 ± 3.7**	8.4 ± 1.0**	40.3**	Prospective randomised trial comparing glargine/metformin + exenatide (*n *= 16) vs. glargine/metformin + sitagliptin (*n *= 16) vs. glargine/metformin (*n *= 16) Follow up, 4 weeks HbA1c decreased by 1.8% with glargine/metformin + exenatide vs. 1.2% with glargine/metformin PPG excursions were substantially reduced with glargine/metformin + exenatide compared with glargine/metformin Weight decreased by 0.9 kg with glargine/metformin + exenatide, increased by 0.4 kg with glargine/metformin Insulin dose did not change with glargine/metformin + exenatide, increased by 5.6 U/day with glargine/metformin Hypoglycaemia rates were comparable among groups
Nayak et al. [20] (2010)	57.5 ± 10.1††	11.4 ± 6.0††	43.2 ± 6.7††	8.8 ± 1.7††	144 ± 90††	Prospective non-comparative trial on exenatide in insulin-treated obese patients Follow up, 12 months (completed 12 months, *n *= 57)\ No change in HbA1c Weight decreased by 12.8 ± 7.5 kg after 12 months Insulin dose decreased to 55 ± 53 U/day after 12 months
Yoon et al. [21] (2009)	56.4 ± 9.4	> 15 years in 77% of included patients	40.4 ± 8.4	8.05 ± 1.47	99.9 ± 90	Retrospective chart analysis of insulin-treated patients receiving adjuvant therapy with exenatide (*n *= 188) Follow up, 18–27 months HbA1c decreased by 0.54 ± 1.4% after 18–27 months of exenatide Weight significantly declined with increasing EXE treatment duration Prandial insulin decreased by 55.7 ± 56.8% after 18–27 months of exenatide
Sheffield et al. [22] (2008)	60 ± 10	15.1 ± 8.1	39.0 ± 11.9	8.25 ± 1.55	63 ± 49	Retrospective chart review to examine insulin-treated patients who received exenatide (*n *= 134) Follow up, 12 months HbA1c decreased by 0.9% Weight decreased by 5.2 kg Basal insulin dose did not change Bolus insulin dose decreased by 9 units/day (45% discontinued premeal insulin)
John et al. [23] (2007)	61 ± 9.4	14 ± 8.5	39.3 ± 7.6	7.8 ± 1.4	51 ± 46	Retrospective chart review of EXE added to insulin, a TZD, a meglitinide, and/or an alpha-glucosidase inhibitor (*n *= 93) Mean follow up, 150 days The average insulin dose decreased to 42 ± 44 U/day; prandial insulin doses decreased from 12 ± 16 to 4 ± 13 U/day Basal insulin dose did not change
Viswanathan et al. [24] (2007)	NR	NR	43.4 ± 1.3‡‡	7.9%‡‡	NR	Retrospective chart analysis on insulin-treated obese patients who started and used exenatide continuously (*n *= 38) or started and then discontinued EXE (*n *= 14) Mean follow up, 26 weeks HbA1c decreased by 0.6 ± 0.21% in the group of continuous exenatide users Weight decreased by 6.46 ± 0.8 kg in the group of continuous exenatide users and increased by 2.4 ± 0.6 kg in the group that discontinued exenatide use Insulin dosage decreased for rapid-acting and mixed insulins in the group of continuous exenatide users

EXE, exenatide BID; GLAR, insulin glargine; NR, not reported; PBO, placebo; PPG, postprandial glucose; T2DM, type 2 diabetes mellitus; TZD, thiazolidinedione.

* Data are mean ± SD.

†Baseline values in the glargine + exenatide group.

‡Baseline values are for all patients in the trial (i.e. exenatide + glargine and glargine + exenatide).

§Reported as total insulin daily dose per body weight (units per kg) in the glargine + exenatide group only.

¶Exenatide + insulin patients only; includes those who continued insulin at exenatide initiation (*n *= 1257) and those who started insulin after exenatide initiation (*n *= 664).

* *Baseline values in the glargine/metformin + exenatide group.

††Baseline values of all audited patients (*n *= 160).

‡‡Baseline values are for all patients in the trial (insulin-treated obese patients who started and used exenatide continuously [*n *= 38] or started and then discontinued exenatide [*n *= 14]).

**Table 2:** Dose titration regimens for basal insulins

Trial Acronym & Reference	Frequency of Insulin Adjustments	Monitored Variable	Measured FPG Level (mg/dl [mmol/l])	Change in Insulin Dosage (U/day)
*Tested with twice-daily exenatide*				
Buse et al. [16]*	Weekly for 5 weeks then at least every other week	Mean FPG of prior 3–7 days	> 180 (> 10) 140–179 (7.8–9.9)† 120–139 (6.7–7.7)† 100–119 (5.6–6.6)† 73–99 (4.1–5.5)	↑ 8 ↑ 6 ↑ 4 ↑ 2 No change
		At least one FPG value since last assessment At least one FPG value since last assessment	56–72 (3.1–4.0) < 56 (< 4.0)	↓ 2 ↓ 4
*Not tested with twice-daily exenatide*				
INITIATE‡ Raskin et al. [43]	Weekly for 12 weeks and then every 2 weeks thereafter	FPG on 3 preceding days	> 180 (> 10) 141–180 (7.8–10) 111–140 (6.2–7.8) 80–110 (4.4–6.2) < 80 (< 4.4)	↑ 6–8† ↑ 4–6† ↑ 2–4† No change ↓ 2
INSIGHT§ Gerstein et al. [41]	Daily	Daily FPG	> 99 (> 5.5)	↑ 1
LANMET¶ Yki-Jarvinen et al. [42]	Varied based on FPG readings	FPG on 3 consecutive mornings	> 180 (> 10) 99–180 (5.5–10)	↑ 4 ↑ 2
4-T** Holman et al. [9]	Not specified	Twice-daily glucose	*Fasting target:* 72–99 mg/dl (4.0–5.5 mmol/l) *Two-hourpostprandial target:*90–126 mg/dl (5.0–7.0 mmol/l)	↑ 10% or 4 U (whichever is greater) if the mean glucose reading for a given time point is > 72 mg/dl (> 4.0 mmol/l) above upper end of range†† Otherwise, ↑ 5% or 4 U (whichever is greater)†† ↓ By 10% or 4 U (whichever is the greater) in the presence of grade 3 hypoglycaemia or mean glucose readings < 56 mg/dl (< 3.1 mmol/l)†† Otherwise, ↓ 5% or 2 U (whichever is greater)††
Rosenstock et al. [11]‡‡	Weekly for 12 weeks and approximately every 3 weeks thereafter	FPG on 3 consecutive mornings	> 180 (> 10) 164–180 (9.1–10) 145–163 (8.1–9.0) 127–144 (7.1–8.0) 109–126 (6.1–7.0) 73–108 (4.1–6.0)	↑ 12 ↑ 8§§ ↑ 6§§ ↑ 4§§ ↑ 2 No change
		One FPG value One FPG value	56–72 (3.1–4.0) < 56 (< 3.1)	↓ 2 ↓ 4

FPG, fasting plasma glucose; ↑, increase by; ↓, decrease by.

* Modified from Treat-to-Target protocol [10]. Trial compared glargine + exenatide vs. glargine + placebo. Insulin starting dosage was 10 U/day. Final mean FPG was 117 mg/dl (6.5 mmol/l) in the glargine + exenatide group and 118 mg/dl (6.6 mmol/l) in the glargine + placebo group.

†The increase in daily dose should not exceed more than 10 U/day or 10% of the current daily dose, whichever is greater.

‡Trial compared glargine vs. biphasic insulin aspart 70/30; final mean FPG was 117 mg/dl (6.5 mmol/l) in the glargine group.

§Trial compared glargine vs. conventional therapy; patients started with 10 units of insulin per day.

¶Trial compared glargine + metformin vs. NPH + metformin. The initial bedtime insulin dose was 10 U for all patients who were using metformin alone, and 20 U if the patients had used both sulfonylurea and metformin and sulfonylurea was stopped, as was mandated by the study design. Final mean FPG was 103 mg/dl (5.7 mmol/l) in the glargine group.

* *Trial compared detemir vs. biphasic aspart vs. prandial insulin. Starting doses of insulin according to the following formulas: for men, [(FPG [mmol/l] − 5) × 2] × (weight [kg] ÷ [14.3 × height (m) − height (m)]; for women, [(FPG [mmol/l] − 5) × 2] × (weight [kg] ÷ (13.2 × height [m]) − height [m]). Final mean FPG was 112 mg/dl (6.2 mmol/l).

††The trial-management system suggested changes in insulin dosages according to the following rules. Maintain prebedtime insulin doses if more than 2/3 of prebreakfast and pre-evening meal glucose readings are within range. Increase prebedtime insulin dose (when no hypoglycaemia) if more than 1/3 of prebreakfast meal glucose readings remain high. Add a prebreakfast insulin injection if glucose readings are at target before breakfast, but not before the evening meal and nocturnal hypoglycaemia limits further prebedtime insulin dose increases. Decrease insulin doses in the presence of: (i) any Grade 2 or 3 hypoglycaemic episode at relevant time points; and (ii) mean glucose readings< 70 mg/dl (< 3.9 mmol/l) at relevant time points.

‡‡Trial compared detemir vs. glargine. Basal insulin was initiated at 12 U/day. Final mean FPG was 131 mg/dl (7.27 mmol/l) in the once-daily detemir group and 126 mg/dl (6.98 mmol/l) in the glargine group.

§§Add 2 units if no response to previous insulin adjustment; no response is defined as occurring if the average self-monitored plasma glucose level is increased and/or within the same range as the last contact.

In the trial, adult patients with type 2 diabetes on insulin glargine with or without metformin or pioglitazone (or both agents) were randomised to either exenatide twice daily (*n* = 138; HbA1c, 8.32 ± 0.85%; body mass index, 33.8 ± 5.8 kg/m^2^; duration of disease, 12 ± 7 years) or matching placebo (*n* = 123; HbA1c, 8.50 ± 0.96%; body mass index, 33.1 ± 6.2 kg/m^2^; duration of disease, 12 ± 7 years) for 30 weeks. (One participant in each arm did not receive any study drugs, so the analyses were performed on 137 and 122 participants respectively). At randomisation, participants with HbA1c > 8.0% continued to receive their current insulin glargine dose, whereas those with HbA1c ≤ 8.0% decreased their dose by 20% to minimise the risk of hypoglycaemia. Exenatide twice daily (5 μg b.i.d. for 4 weeks followed by 10 μg b.i.d.) or placebo was then added, while the basal insulin doses were maintained. At 5 weeks, insulin glargine was actively titrated to a target FPG < 100 mg/dl (< 5.6 mmol/l) according to a modified form of the Treat-to-Target protocol (10). Participants continued their prestudy doses of metformin and pioglitazone throughout the study period.

**Figure 2:**
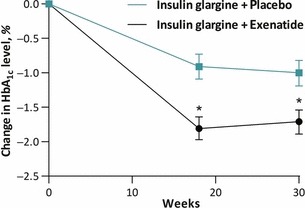
Effects of exenatide twice daily plus optimized insulin glargine versus optimized insulin glargine on HbA1c (16)

**Figure 3:**
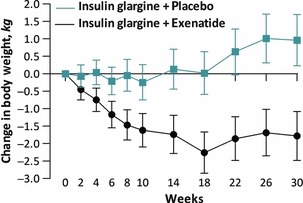
Effects of exenatide twice daily plus optimized insulin glargine versus optimized insulin glargine on body weight (16)

Both treatment groups had nearly identical reductions in FPG at 30 weeks [–1.6 vs. –1.5 mmol/l (−28.8 vs. −27 mg/dl)], indicating similar responses to the insulin adjustment algorithm. Final mean FPG levels from self-monitored blood glucose measurements were 117 mg/dl (6.5 mmol/l) and 118 mg/dl (6.6 mmol/l) in the exenatide and placebo groups respectively. Reductions in all other glycaemic parameters were greater in the exenatide group than in the placebo group, including HbA1c (−1.74% vs. −1.04%; p < 0.001; Figure 2), proportion of patients reaching HbA1c ≤ 7% (60% vs. 35%; p < 0.001) and proportion of patients reaching HbA1c ≤ 6.5% (40% vs. 12%; p < 0.001). At 30 weeks, self-monitored blood glucose values were also lower at all non-fasting time points in the exenatide group (p < 0.001), as were morning and evening postprandial glucose excursions. Insulin dosage increased from baseline in both groups, but the increase was greater in the placebo group (20 vs. 13 units per day; p = 0.030). Despite the increase in insulin dose, mean body weight decreased by 1.78 kg in the exenatide group, whereas it increased by 0.96 kg in the placebo group (p < 0.001) (Figure 3). Thus, the combination of exenatide and titrated insulin glargine provided greater glycaemic control than basal insulin alone, and did so with modest weight loss and no increased risk of hypoglycaemic events.

More exenatide recipients than placebo recipients discontinued the study because of adverse events [13 (9%) vs. 1 (1%), respectively; p < 0.010]. Serious adverse events were evenly distributed between groups, and none was experienced by more than two participants in either group. One death (resulting from myocardial infarction) occurred in the placebo group. No treatment-emergent pancreatitis, acute renal failure or cancer occurred. The number of hypoglycaemic events per participant per year did not differ significantly between the two groups, although nausea (41% vs. 8%), diarrhoea (18% vs. 8%), vomiting (18% vs. 4%), headache (14% vs. 4%) and constipation (10% vs. 2%) were more prevalent in the exenatide group.

Eight observational studies also examined insulin–exenatide combination therapy in patients with type 2 diabetes (17–24) (Table 1). Seven of these (17–19,21–24) were consistent with the Buse et al. study (16) in showing reductions in blood glucose in patients who had added exenatide to insulin therapy. All showed reductions in patient weight. In addition, several of the observational studies reported a concomitant reduction in insulin dose. The incidence of reported severe hypoglycaemia according to the definitions within each study was low. One of the largest of these studies is an example of one of these observational studies. The Association of British Clinical Diabetologists (ABCD) nationwide exenatide audit collected data across 126 centres in the UK (18,36). In total, 1257 patients in the ABCD study received exenatide treatment as add-on to continuing insulin therapy (primarily in combination with oral antidiabetic agents). Patients treated with a combination of exenatide twice daily and insulin experienced a mean HbA1c reduction of 0.51%, weight reduction of 5.8 kg, insulin dose reduction of 42 units per day and insulin discontinuation rate of 16.6%.

The retrospective chart review by Yoon et al. (21) provided greater detail, especially regarding temporal effects, than many other studies. This university-based study examined 188 patients with type 2 diabetes who had received insulin plus adjunctive exenatide for a mean duration of 350 ± 208 days (mean baseline parameters: HbA1c, 8.05 ± 1.47%; age, 56 ± 9 years; body mass index, 40.4 ± 8.4 kg/m^2^; and total daily insulin dose, 99.9 ± 90.0 U). The last observations in four prespecified time intervals (0–6, 6–12, 12–18 and 18–27 months) were used in the analyses. Mean changes in HbA1c were –0.66 ± 1.54% at 0–6 months (p < 0.001); −0.55 ± 1.4% at 6–12 months (p < 0.001); −0.54 ± 1.83% at 12–18 months (p = 0.019); and −0.54 ± 1.37% at 18–27 months (p = 0.020). Weight decreased significantly at each time point (2.4–6.2 kg across the four time points). The total mean daily dose of insulin was decreased in all patients at the 0 to 6-month (−18.0 ± 49.9 U; p < 0.001) and 6 to 12-month (−14.8 ± 35.3 U; p < 0.001) intervals. Significant decreases were observed in the daily prandial insulin doses for all four intervals, but basal insulin doses did not change. In the study, 26% of patients discontinued exenatide because of adverse events. The most common adverse events were mild nausea and vomiting. One patient (0.4%) experienced mild hypoglycaemia.

## Practical considerations

Exenatide twice daily has been available in the USA since 2005 for treatment of hyperglycaemia in type 2 diabetes. In the US, it was initially indicated as an add-on therapy after the failure of oral agents and, then, as monotherapy (32). In addition, several studies demonstrated the advantages of initiating exenatide twice daily prior to insulin after oral agent failure with respect to weight gain and, potentially, hypoglycaemia (34,37,38). Thus, the data described in the prior section expands the clinical considerations for exenatide twice daily still further by including patients who already require basal insulin. Exenatide twice daily added to insulin glargine has been approved in the US for the treatment of type 2 diabetes. It is approved in combination with any basal insulin in the EU.

### Patient selection

Selection criteria for patients who may be appropriate for the combination of basal insulin and exenatide twice daily can be informed by data from the randomised trial of Buse et al. (16), as well as the previously described observational studies (17–24). As patients in these studies had initial mean HbA1c values ranging from 7.8 to 9.52% (Table 1), the complementary effects of basal insulin plus exenatide twice daily appeared to be observed across a wide range of HbA1c values. This may be consistent with the observations of Monnier et al. (39), who showed that FPG (primarily targeted by basal insulin) is the major contributor to HbA1c when HbA1c values are very elevated, whereas postprandial glucose (primarily targeted by twice-daily exenatide) is the major contributor to overall hyperglycaemia at lower HbA1c levels. A follow-up report to the Buse et al. study (16) also found that patients treated with exenatide maintained efficacy across a wide range of durations of type 2 diabetes, i.e. HbA1c reduction was the same in patients with > 15 years of disease as it was in disease of shorter duration (40). Finally, patients on high doses of insulin (e.g. > 0.5 U/kg) exhibited reductions in both HbA1c (16) and insulin use (18,20,21) after the addition of exenatide. Thus, the addition of twice-daily exenatide to titrated insulin glargine may be considered in multiple types of patients with type 2 diabetes that have sub-optimal glycaemic control.

### Avoiding hypoglycaemia

The principal practical challenge in adding exenatide twice-daily therapy for patients already on basal insulin is the risk of hypoglycaemia, coupled with minimal knowledge regarding home glucose levels. In actual practice, many patients perform home glucose monitoring only in the fasting state. FPG, however, is a poor predictor of overall blood glucose control and, in particular, provides no information about postprandial hyperglycaemia, the period when exenatide exerts most of its benefit. Titration of basal insulin frequently does not take into account elevation of postdinner hyperglycaemia, so for a subset of patients, morning FPG may reflect a substantial fall from bedtime levels. As exenatide twice-daily therapy may reduce postprandial glucose readings by more than 100 mg/dl (5.6 mmol/l) and FPG by ∼ 25 mg/dl (1.4 mmol/l) (28), sensible reduction in basal insulin is prudent as a safety measure to avoid hypoglycaemia at the initiation of exenatide therapy.

### Insulin titration

Multiple titration regimens for basal insulin have been examined in clinical trial settings, each targeting FPG to normal or near normal levels (9–11,16,41–43) (Table 2). The regimen used in the Buse et al. study (16) has been tested in combination with twice-daily exenatide in a rigorous prospective clinical trial setting and can therefore be recommended. This simple regimen, a modified version of the Treat-to-Target protocol first described by Riddle et al. (10), titrates basal insulin once weekly for 5 weeks, then at least biweekly thereafter, based upon mean FPG values from the previous 3–7 days. The protocol also mandates down-titration of basal insulin after individual instances of FPG readings that fall below target. Notably, the modified Treat-to-Target regimen in combination with exenatide resulted in mean FPG values under 120 mg/dl with acceptable hypoglycaemia risk (16).

The other basal insulin titration protocols described in Table 2 have not been examined in prospective clinical trials with exenatide, but a variety of observational studies have reported on different types of approaches for insulin–exenatide combination therapy and found good efficacy with little risk of hypoglycaemia (17–24). Based on the results of these reported observations, treating physicians may, therefore, feel comfortable incorporating other regimens that best fit the needs of their patients, provided that they adhere to certain precautions. Exenatide administration should follow the recommended dosing of 5 μg b.i.d. for 4 weeks followed by 10 μg b.i.d. as tolerated. When exenatide is added to insulin, down-titration of the insulin dose should be considered. Buse et al. (16) reduced insulin by 20% for all participants whose entry HbA1c was ≤ 8.0% (16), which likely reduced hypoglycaemia risk. It is important to note that basal insulin titration should be an ongoing process, even after starting exenatide twice daily. In general, regimens that increase insulin on a weekly basis for elevated glucose values and reduce insulin for single low glucose values should provide good efficacy and safety.

Many physicians prefer to reduce the dose of insulin based on FPG rather than HbA1c values. The following observations of exenatide’s effect on fasting glucose may help to guide this approach. Exenatide 10 μg b.i.d. is typically associated with a reduction in mean FPG of approximately 10–11 mg/dl (44–46); however, higher mean reductions have been reported, including reductions of 19 mg/dl (47), 26 mg/dl (34) and 28 mg/dl (48). Reducing insulin by 20% for patients whose fingerstick values are under 120 mg/dl may be prudent. As the effects of exenatide on glucose are rapid (28), up-titration of insulin can begin once stable FPG levels have been achieved after starting exenatide 10 μg b.i.d. The approach used by Buse et al. (16) suggests that this may safely occur 1 week after starting exenatide 10 μg b.i.d.

## Other incretin therapies in combination with basal insulin

Two other GLP-1 receptor agonists are currently available in the USA and the European Union: exenatide once weekly, and liraglutide once daily (49,50). Neither of these agents has been studied as add-on therapy to basal insulin in patients who exhibited inadequate glycaemic control on basal insulin alone (± oral antidiabetic agents), as described in the Buse study (16). Whether the results and recommendations reported here with exenatide twice daily apply to exenatide once weekly or liraglutide, therefore, remains uncertain.

Liraglutide has also been examined in combination with a basal insulin, but the order of addition was different from the Buse study (16), i.e. basal insulin was added to patients who had inadequate glycaemic control on prior liraglutide therapy, rather than *vice versa*. Thus, in one prospective study, 323 patients with type 2 diabetes uncontrolled on metformin plus liraglutide (baseline HbA1c, 7.6%) were randomised to add-on insulin detemir (*n* = 162) or continuation without insulin detemir (*n* = 161). After 26 weeks of therapy, HbA1C changed by −0.5% with insulin detemir and + 0.02% without insulin detemir (p < 0.0001). No major hypoglycaemia occurred and minor hypoglycaemia rates were 0.286 and 0.029 events per participant year with and without insulin detemir (9.2% vs. 1.3%). The HbA1c efficacy reported in this study was therefore likely to be attributable to the insulin detemir.

Three DPP4 inhibitors have been studied as add-on therapy to insulin (51–53). Reductions in HbA1c were in the range of 0.4–0.7%. These studies did not use a forced titration of basal insulin, although changes in insulin doses within specified limits and discontinuation or rescue therapy for hyperglycaemia were often permitted. Therefore, without further clinical trials, these studies again cannot be directly compared with the Buse study (16), in which insulin was adjusted to fasting targets and the exenatide effects could be attributed to the effects on prandial glucose levels.

## Conclusions

In patients with type 2 diabetes, clinical studies indicate that adding exenatide twice daily to basal insulin results in robust reductions in HbA1c, modest weight reduction, no significant increase in hypoglycaemia and (in observational studies) a possible reduction in insulin dose. The combination appears to be efficacious across a wide range of patient types. Several simple titration regimens have been provided in this review for consideration, along with recommendations for down-titration of insulin in patients with minimal elevated FPG levels (or HbA1c < 8%)

## References

[1] American Diabetes Association (2012). Standards of medical care in diabetes – 2012. Diabetes Care.

[2] International Diabetes Federation (2011). Treatment Algorithm for People with Type 2 Diabetes. http://www.idf.org/treatment-algorithm-people-type-2-diabetes.

[3] Inzucchi SE, Bergenstal RM, Buse JB (2012). Management of hyperglycemia in type 2 diabetes: a patient-centered approach: Position Statement of the American Diabetes Association (ADA) and the European Association for the Study of Diabetes (EASD). Diabetes Care.

[4] Handelsman Y, Mechanick JI, Blonde L (2011). American Association of Clinical Endocrinologists Medical Guidelines for Clinical Practice for developing a diabetes mellitus comprehensive care plan. Endocr Pract.

[5] UK Prospective Diabetes Study (UKPDS) Group (1998). Intensive blood-glucose control with sulphonylureas or insulin compared with conventional treatment and risk of complications in patients with type 2 diabetes (UKPDS 33). UK Prospective Diabetes Study (UKPDS) Group. Lancet.

[6] Turner RC, Cull CA, Frighi V, Holman RR (1999). Glycemic control with diet, sulfonylurea, metformin, or insulin in patients with type 2 diabetes mellitus: progressive requirement for multiple therapies (UKPDS 49). UK Prospective Diabetes Study (UKPDS) Group. JAMA.

[7] Koro CE, Bowlin SJ, Bourgeois N, Fedder DO (2004). Glycemic control from 1988 to 2000 among U.S. adults diagnosed with type 2 diabetes: a preliminary report. Diabetes Care.

[8] Hermansen K, Davies M, Derezinski T (2006). A 26-week, randomized, parallel, treat-to-target trial comparing insulin detemir with NPH insulin as add-on therapy to oral glucose-lowering drugs in insulin-naive people with type 2 diabetes. Diabetes Care.

[9] Holman RR, Thorne KI, Farmer AJ (2007). Addition of biphasic, prandial, or basal insulin to oral therapy in type 2 diabetes. N Engl J Med.

[10] Riddle MC, Rosenstock J, Gerich J (2003). The treat-to-target trial: randomized addition of glargine or human NPH insulin to oral therapy of type 2 diabetic patients. Diabetes Care.

[11] Rosenstock J, Davies M, Home PD (2008). A randomised, 52-week, treat-to-target trial comparing insulin detemir with insulin glargine when administered as add-on to glucose-lowering drugs in insulin-naive people with type 2 diabetes. Diabetologia.

[12] Cryer PE (2002). Hypoglycaemia: the limiting factor in the glycaemic management of Type I and Type II diabetes. Diabetologia.

[13] Hermansen K, Mortensen LS (2007). Bodyweight changes associated with antihyperglycaemic agents in type 2 diabetes mellitus. Drug Saf.

[14] Ismail-Beigi F, Moghissi E, Tiktin M (2011). Individualizing glycemic targets in type 2 diabetes mellitus: implications of recent clinical trials. Ann Intern Med.

[15] Genuth S, Ismail-Beigi F (2012). Clinical implications of the ACCORD trial. J Clin Endocrinol Metab.

[16] Buse JB, Bergenstal RM, Glass LC (2011). Use of twice-daily exenatide in basal insulin-treated patients with type 2 diabetes: a randomized, controlled trial. Ann Intern Med.

[17] Levin PA, Mersey JH, Zhou S, Bromberger LA (2011). Clinical outcomes using long-term combination therapy with insulin glargine and exenatide In patients with type 2 diabetes. Endocr Pract.

[18] Thong KY, Jose B, Sukumar N (2011). Safety, efficacy and tolerability of exenatide in combination with insulin in the Association of British Clinical Diabetologists nationwide exenatide audit. Diabetes Obes Metab.

[19] Arnolds S, Dellweg S, Clair J (2010). Further improvement in postprandial glucose control with addition of exenatide or sitagliptin to combination therapy with insulin glargine and metformin: a proof-of-concept study. Diabetes Care.

[20] Nayak UA, Govindan J, Baskar V (2010). Exenatide therapy in insulin-treated type 2 diabetes and obesity. QJM.

[21] Yoon NM, Cavaghan MK, Brunelle RL, Roach P (2009). Exenatide added to insulin therapy: a retrospective review of clinical practice over two years in an academic endocrinology outpatient setting. Clin Ther.

[22] Sheffield CA, Kane MP, Busch RS (2008). Safety and efficacy of exenatide in combination with insulin in patients with type 2 diabetes mellitus. Endocr Pract.

[23] John LE, Kane MP, Busch RS, Hamilton RA (2007). Expanded use of exenatide in the management of type 2 diabetes. Diabetes Spectrum.

[24] Viswanathan P, Chaudhuri A, Bhatia R (2007). Exenatide therapy in obese patients with type 2 diabetes mellitus treated with insulin. Endocr Pract.

[25] Kendall DM, Cuddihy RM, Bergenstal RM (2009). Clinical application of incretin-based therapy: therapeutic potential, patient selection and clinical use. Eur J Intern Med.

[26] Defronzo RA (2009). Banting Lecture. From the triumvirate to the ominous octet: a new paradigm for the treatment of type 2 diabetes mellitus. Diabetes.

[27] Drucker DJ (2006). The biology of incretin hormones. Cell Metab.

[28] Kolterman OG, Buse JB, Fineman MS (2003). Synthetic exendin-4 (exenatide) significantly reduces postprandial and fasting plasma glucose in subjects with type 2 diabetes. J Clin Endocrinol Metab.

[29] Kolterman OG, Kim DD, Shen L (2005). Pharmacokinetics, pharmacodynamics, and safety of exenatide in patients with type 2 diabetes mellitus. Am J Health Syst Pharm.

[30] Cervera A, Wajcberg E, Sriwijitkamol A (2008). Mechanism of action of exenatide to reduce postprandial hyperglycemia in type 2 diabetes. Am J Physiol Endocrinol Metab.

[31] Cersosimo E, Gastaldelli A, Cervera A (2011). Effect of exenatide on splanchnic and peripheral glucose metabolism in type 2 diabetic subjects. J Clin Endocrinol Metab.

[32] (2011). Byetta.

[33] Bunck MC, Corner A, Eliasson B (2010). One-year treatment with exenatide vs. insulin glargine: effects on postprandial glycemia, lipid profiles, and oxidative stress. Atherosclerosis.

[34] Heine RJ, Van Gaal LF, Johns D (2005). Exenatide versus insulin glargine in patients with suboptimally controlled type 2 diabetes: a randomized trial. Ann Intern Med.

[35] Macconell L, Brown C, Gurney K, Han J (2012). Safety and tolerability of exenatide twice daily in patients with type 2 diabetes: integrated analysis of 5594 patients from 19 placebo-controlled and comparator-controlled clinical trials. Diabetes Metab Syndr Obes.

[36] Ryder REJ, Thong KY, Cull ML (2010). The Association of British Clinical Diabetologists (ABCD) nationwide exenatide audit. Practical Diabetes International.

[37] Davies MJ, Donnelly R, Barnett AH (2009). Exenatide compared with long-acting insulin to achieve glycaemic control with minimal weight gain in patients with type 2 diabetes: results of the Helping Evaluate Exenatide in patients with diabetes compared with Long-Acting insulin (HEELA) study. Diabetes Obes Metab.

[38] Barnett AH, Burger J, Johns D (2007). Tolerability and efficacy of exenatide and titrated insulin glargine in adult patients with type 2 diabetes previously uncontrolled with metformin or a sulfonylurea: a multinational, randomized, open-label, two-period, crossover noninferiority trial. Clin Ther.

[39] Monnier L, Lapinski H, Colette C (2003). Contributions of fasting and postprandial plasma glucose increments to the overall diurnal hyperglycemia of type 2 diabetic patients: variations with increasing levels of HbA(1c). Diabetes Care.

[40] Rosenstock J, Shenouda SK, Bergenstal RM (2012). Baseline factors associated with glycemic control and weight loss when exenatide twice daily Is added to optimized insulin glargine in patients with type 2 diabetes. Diabetes Care.

[41] Gerstein HC, Yale JF, Harris SB (2006). A randomized trial of adding insulin glargine vs. avoidance of insulin in people with Type 2 diabetes on either no oral glucose-lowering agents or submaximal doses of metformin and/or sulphonylureas. The Canadian INSIGHT (Implementing New Strategies with Insulin Glargine for Hyperglycaemia Treatment) Study. Diabet Med.

[42] Yki-Jarvinen H, Kauppinen-Makelin R, Tiikkainen M (2006). Insulin glargine or NPH combined with metformin in type 2 diabetes: the LANMET study. Diabetologia.

[43] Raskin P, Allen E, Hollander P (2005). Initiating insulin therapy in type 2 Diabetes: a comparison of biphasic and basal insulin analogs. Diabetes Care.

[44] Buse JB, Henry RR, Han J (2004). Effects of exenatide (exendin-4) on glycemic control over 30 weeks in sulfonylurea-treated patients with type 2 diabetes. Diabetes Care.

[45] DeFronzo RA, Ratner RE, Han J (2005). Effects of exenatide (exendin-4) on glycemic control and weight over 30 weeks in metformin-treated patients with type 2 diabetes. Diabetes Care.

[46] Kendall DM, Riddle MC, Rosenstock J (2005). Effects of exenatide (exendin-4) on glycemic control over 30 weeks in patients with type 2 diabetes treated with metformin and a sulfonylurea. Diabetes Care.

[47] Moretto TJ, Milton DR, Ridge TD (2008). Efficacy and tolerability of exenatide monotherapy over 24 weeks in antidiabetic drug-naive patients with type 2 diabetes: a randomized, double-blind, placebo-controlled, parallel-group study. Clin Ther.

[48] Zinman B, Hoogwerf BJ, Duran Garcia S (2007). The effect of adding exenatide to a thiazolidinedione in suboptimally controlled type 2 diabetes: a randomized trial. Ann Intern Med.

[49] (2011). Victoza.

[50] (2012). Bydureon.

[51] Barnett AH, Charbonnel B, Donovan M (2012). Effect of saxagliptin as add-on therapy in patients with poorly controlled type 2 diabetes on insulin alone or insulin combined with metformin. Curr Med Res Opin.

[52] Fonseca V, Schweizer A, Albrecht D (2007). Addition of vildagliptin to insulin improves glycaemic control in type 2 diabetes. Diab tologia.

[53] Vilsboll T, Rosenstock J, Yki-Jarvinen H (2010). Efficacy and safety of sitagliptin when added to insulin therapy in patients with type 2 diabetes. Diabetes Obes Metab.

